# Causality of occupational exposure on rheumatoid arthritis and ankylosing spondylitis: a two-sample mendelian randomization study

**DOI:** 10.3389/fimmu.2023.1223810

**Published:** 2023-10-02

**Authors:** Kai Du, Chen-Yu Zhang, Ao Li, Jia-Ze Hu, Ren Guo, Shu-Ming Li

**Affiliations:** ^1^ Graduate School, Beijing University of Chinese Medicine, Beijing, China; ^2^ Department of Computer Science and Technology, Changchun Normal University, Changchun, China; ^3^ Department of Pain Medicine, Beijing Hospital of Traditional Chinese Medicine, Capital Medical University, Beijing, China

**Keywords:** occupational exposure, Mendelian randomization, rheumatoid arthritis, ankylosing spondylitis, rheumatism

## Abstract

**Objective:**

This study aimed to explore the potential causal link between three specific types of occupational exposure on rheumatoid arthritis (RA) and ankylosing spondylitis (AS).

**Method:**

A Two-sample Mendelian randomization (TSMR) analysis, comprising univariate MR (UVMR) and multivariate MR (MVMR) analyses, was performed to investigate the potential causal association between three types of occupational exposures, jobs involving mainly walking or standing (JWS), jobs involving heavy manual or physical work (JMP), and jobs involving shift work(JSW) on RA and AS. Genetic variants for genome-wide association studies (GWAS) of occupational exposure and AS were obtained from the UK Biobank. GWAS summary data for RA were obtained from FinnGen Biobank analysis. For UVMR, six methods of Inverse Variance Weighted (IVW), MR-Egger, Weighted Mode, Weighted Median, Simple Mode, MR pleiotropy residual sum, and outlier (MR-PRESSO) were used for the analysis. The MVMR was analyzed using the IVW model as well as the MR-Egger model.

**Results:**

The UVMR suggested no causal relationship between the three occupational exposure and RA [IVW: P=0.59,0.21,0.63] or AS [IVW: P=0.43,0.57,0.04], as did the bidirectional MR [IVW: P=0.73,0.70,0.16], [IVW: P=0.65,0.68,0.74]. Although unadjusted MVMR suggested a causal relationship between JMP and AS [IVW: OR = 1.01, 95% CI = 1.00- 1.02, p = 0.02], the adjusted MVMR denied this relationship and concluded that there was no causal relationship between the other occupational exposure and either RA or AS.

**Conclusion:**

Our MR analysis did not establish a direct causal relationship between certain occupational exposures and either RA or AS.

## 1 Introduction

With social and economic development and noteworthy changes in people’s working environment and conditions, the impact of occupational exposure on personal health is becoming ever more apparent, going beyond physical health to include mental health. For instance, prolonged sedentary behavior and overwork have been linked to an increased prevalence of obesity and cardiovascular diseases (1). Furthermore, increased work pressure and psychological burden are associated with a higher likelihood of mental health problems such as depression and anxiety (2). Extended maintenance of the same body position or posture during work, such as walking, standing, or bending, can easily cause physical fatigue, muscle tension, joint pain, and other occupational diseases (3). Manual labor is one of human history’s oldest forms and is still required in many industries. However, harsh working environments and chronic physical work can lead to numerous motion system and musculoskeletal disorders (4). Furthermore, excessive working hours and unreasonable working methods can disrupt individuals’ biological clocks, resulting in sleep problems and other related health issues, as with shift work (5).

Linkage appears to exist between occupational factors and rheumatic diseases (6–11). Rheumatism, a complex autoimmune disorder, can affect various body systems and, when primarily impacting the joints, can manifest as either rheumatoid arthritis (RA) or ankylosing spondylitis (AS). These chronic, progressive arthritis typically cause joint swelling, pain, and stiffness and can impair the function of the affected joints and surrounding tissues (12). RA is more prominent in women than men and is most often seen in individuals between 30 and 50, with multi-joint involvement and symmetrical distribution of the affected joints, particularly in the hands, wrists, knees, and feet. AS, on the other hand, is more common among men and younger individuals (20-30 years old) and is characterized by stiffness and pain in the spine and cervical spine, as well as potential curvature of the spine, which can impair activities of daily living (13–16). Though the etiology of RA and AS is not fully understood, a combination of genetic, environmental, and immune factors is believed to contribute to their pathogenesis, with occupational exposure thought to play a role in their occurrence. Few studies have suggested that occupational walking or standing can directly contribute to the development of RA, and lower extremity fatigue associated with it may be linked to RA incidence (17). As for strenuous occupational and physical activity, its relationship to the progression of RA and AS is contested, while some findings suggest a correlation, and others hold the opposite opinion (18, 19). Fewer studies have assessed the relationship between occupational shift work on RA or AS, with some suggesting that shift work may be correlated with RA progression. However, it has also been suggested that the opposite is true and therefore more data is needed (20–22). Observational studies may indicate a relationship between occupational exposure and RA or AS. However, such studies face several issues, including uncontrolled variables, inaccurate data, potential confounders, and reverse causality, leading to inconsistent, non-generalizable, or contradictory findings. As such, the causal relationship between occupational exposure to RA and AS remains inconclusive, and our understanding of the relationship between them is limited.

Mendelian randomization (MR) is a statistical method for assessing causality using genetic variants as naturally randomly assigned instrumental variables (IVs). This avoids confounding bias due to the interaction between observed characteristics and potential confounders. It, therefore, provides higher confidence in the results than observational studies (23). Attributed to the randomization that mimics the splitting process, MR can reduce the influence of study results by chance confounding factors. Also, MR can avoid reversing causality interference in chronological order since the genotypes of the phenotypes of interest are not likely to be altered (24). MR studies are revolutionary because they can be used to explore the causal relationships, mediators, and potential mechanisms of action between occupational exposure and RA with AS without the need for large-scale surveys and data collection, and their results can also reflect the current social situation. In MR studies, single nucleotide polymorphisms (SNPs) were used as IVs to identify possible causality between exposure and outcome.

To assess whether occupational exposure is a causal risk factor for RA and AS, we utilized a Two-Sample Mendelian randomization (TSMR), which included univariate Mendelian randomization (UVMR) and multivariate Mendelian randomization (MVMR) to determine the causal relationship between RA with AS and occupational exposure in the current study, which utilized summary statistics from genome-wide association studies (GWAS). This study follows the "Strengthening the Reporting of Observational Studies in Epidemiology using Mendelian Randomization"(STROBE-MR) checklist.

## 2 Materials and methods

### 2.1 Study design and basic assumptions of MR

**Figure 1:**
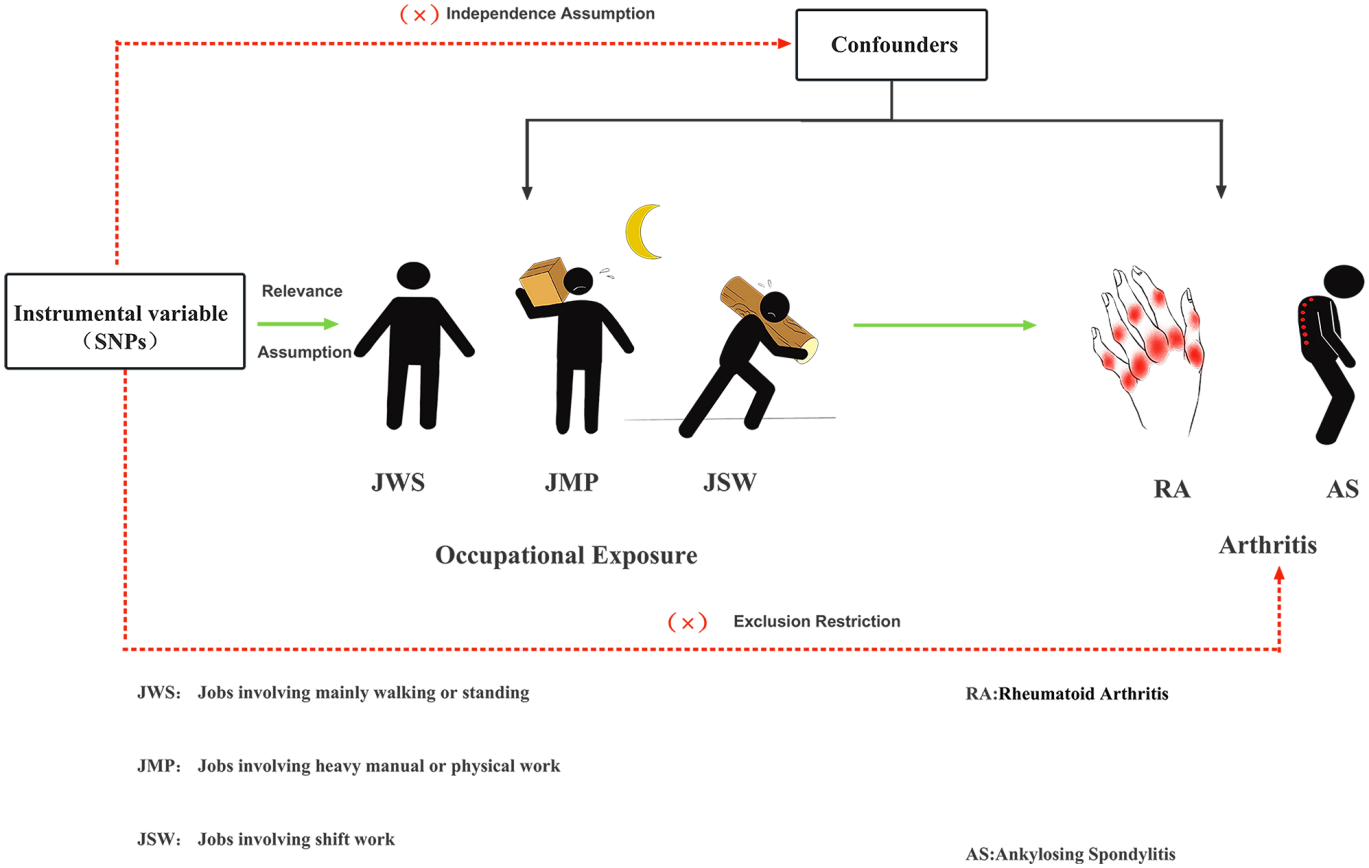
Three major hypotheses of MR to investigate the causal relationship between occupational exposure and RA with AS.

This TSMR study aimed to investigate the potential causal relationship between three work-related phenotypes: jobs involving mainly walking or standing (JWS), jobs involving heavy manual or physical work (JMP), and jobs involving shift work (JSW), on RA and AS. We performed UVMR and MVMR to obtain more accurate results, respectively. The MR study is based on three hypotheses. First, IVs are closely related to occupational exposure. Second, IVs were not associated with confounders between occupational exposure to RA and AS. Finally, IVs had no direct effect on RA and AS, and their impact on them could only be manifested through occupational exposure. As shown in **Figure 1**, an experimental design was then applied to the MR study.

### 2.2 Data sources and populations

This study used pooled-level results from the GWAS, which included three exposure phenotypes associated with occupational exposures (JWS, JMP, JSW), disease outcomes (RA and AS), and covariates that acted as risk factors (smoking, obesity, depression, infections, vitamin D deficiency), all of which were obtained from the IEU Open GWAS (https://gwas.mrcieu.ac.uk/).The work-related data provided by the UK Biobanking (UKB) project is a GWAS dataset covering approximately 950,000 SNPs in both males and females, with populations originating from Europe (25). The work-related phenotypes involved JWS (sample size = 263,556), JMP (sample size = 263,615), and JSW (sample size = 263,315). Informed consent was given, and participants completed thorough questionnaires from UKB to gather relevant information on their demographics, healthy beings, and other traits. Included in this was a touchscreen questionnaire that asked about occupational demands (job always/usually/sometimes/rarely ‘involves mainly walking or standing’ or ‘involves heavy manual or physical work’ or ‘involves shift work’), meaning that all questions were self-reported categorical variables (26). It is important to acknowledge that while self-reporting has some limitations, such as potential recall bias or misclassification, the use of standardized questionnaires and clear instructions to reduce reporting bias and improve data quality. Data for RA patients of 218,790 were obtained from FinnGen biobank (case=4,594, control=214,196), and finally, we got data for 337,159 AS patients (case=968, control=336,191) (27). Given the complexity of disease etiology and the interactions between various risk factors, SNPs as proxies for occupational exposures may directly influence RA and AS risk through factors such as obesity, smoking, depression, infections, and vitamin D deficiency, and we therefore added these risk factors to the MVMR to correct for possible potential bias (28). Specifically, we included 98,697 people with obesity (case=32,858, control=65,839), 607,291 people with smoking (case=311,629, control=321,173), 322,580 people with depression (case=113,769, control=208,811), 218,637 people with infection (case=20,977, control=197,660), and 209,789 people with Vitamin D deficiency (case=182, control= 209,607). Our detailed data are in **Supplementary Tables 1-5**.

### 2.3 Selection of the genetic instruments

To ensure the validity of our genetic IVs and satisfy the three core MR assumptions, we employed a comprehensive set of quality control techniques. Initially, we subjected the genome-wide SNPs to a stringent screening process, applying a threshold of p < 5 × 10^-8^. This rigorous selection aimed to identify highly significant SNPs. However, in cases where the number of eligible SNPs was less than 10, we relaxed the threshold range to p < 5 × 10^-6^ while still adhering to the basic requirements for MR studies (29). We also employed a linkage disequilibrium (LD) clustering approach to refine our results further and eliminate SNPs in strong LD. We utilized a threshold of r2 = 0.001 and kb = 10,000 to ensure accurate and precise clustering. Furthermore, we took additional measures to ensure the consistency and reliability of our IVs. We harmonized the exposure and outcome data to eliminate ambiguous SNPs with inconsistent alleles. Additionally, we excluded SNPs with intermediate allele frequencies, further refining the selection process. It is widely accepted that an F-statistic exceeding 10 indicates a substantial connection between the IV and the exposure. In addition, we computed R², which represents the proportion of phenotypic variation explained by the SNPs in our database. The calculation involved the formula [2 * EAF * (1 - EAF) * beta^2]/(SE^2 * N), where EAF denotes the effect allele frequency, beta is the allele effect value, SE is the standard error, and N is the sample size (30, 31). After meticulously applying these rigorous quality control measures, we identified a set of carefully selected SNPs that served as our final genetic IVs for subsequent MR analysis. These IVs were chosen based on their reliability and adherence to the established criteria. The SNPs screening process for two sets of bidirectional TSMR is shown in **Supplementary Figure 1**.

### 2.4 Two samples Mendelian randomization

Using the TwoSampleMR and MR-PRESSOR R packages in UVMR, and the TwoSampleMR and MendelianRandomization R packages in MVMR, both run in R (version 4.3.0), a software environment for statistical computing and graphics. The TwoSampleMR (version 0.5.6) is an R package that provides many computational methods, extensive published GWAS data, which is extremely flexible. It was used to perform MR Egger, weighted median, inverse variance weighted (IVW), simple mode and weighted mode (32).The MR-PRESSOR package, which allows for the evaluation of horizontal pleiotropy utilizing GWAS statistics, is a widely used method that includes MR-PRESSO global test, MR-PRESSO outlier test and MR-PRESSO distortion test (33).MendelianRandomization (version 0.7.0) is an R package from CRAN, mainly written by Stephen Burgess, which provides a lot of useful features and makes up for the shortcomings of the TwoSampleMR package in some ways (28, 33–35). For MVMR analysis, the TwoSampleMR package was used to get results for Multi-variables IVW model and the MendelianRandomization package was used to get results of Multi-variables MR-Egger, which made our MVMR results more reliable. Utilizing these robust programs has empowered us to explore the etiology of RA and AS, with a specific focus on the impact of occupational exposure on the development of this condition.

The IVW method is a commonly used meta-analysis method that can be used to combine the effects of multiple independent genetic variants on a target factor for a weighted average, resulting in a more accurate estimate of the overall causal effect, with the advantages of reliability and efficiency. MR-Egger is an extension of MR that helps to address the identification and correction of any bias introduced by horizontal pleiotropy, allowing more reliable causal inferences to be made from the data (36). The weighted median can produce consistent results, with approximately half of the instrumental variables invalid (37). MR-PRESSO identifies and removes outliers with p<0.05 and gives corrected causal effects that are used to correct horizontal pleiotropy (33). The results were reported regarding odds ratios (OR) and their corresponding 95% confidence intervals (CI). IVW with an OR greater than 1, 95% CI not passing through 0, and a p-value less than 0.05 was statistically significant. The Bonferroni correction was used to adjust for multiple testing and reduce the risk of false positives in UVMR. The threshold for statistical significance was set at a bilateral p-value (IVW) of less than 0.0083. This correction is necessary because when multiple tests are performed, the likelihood of false positives increases, which helps control this issue. In UVMR, we use IVW as the primary result and the other five methods as the secondary results, and we consider the results reliable when IVW and the other five methods show consistency. In MVMR, when both multivariate IVW model and the multivariate MR-Egger shows a positive result, we consider the result to be reliable. Statistical efficacy was calculated using the mRnd website (https://shiny.cnsgenomics.com/mRnd/). Not adjusted with the same set of covariates in both samples.

### 2.5 Sensitivity analysis

We utilized the Cragg- Donald F-statistic to assess the strength of our IVs and mitigate weak instrument bias. This statistic was calculated as F=beat²/se² (38). Cochran’s Q statistic assessed heterogeneity, and P<0.05 was considered a significant level of heterogeneity. MR Egger and MR-PRESSO were used to determine potential uncorrelated horizontal pleiotropy effects. MR-Egger’s intercept is used to assess multiplicity, and if the intercept term differs significantly from 0(P<0.05), then it indicates that horizontal diversity may exist among these IVs. MR-PRESSO analysis can identify horizontal pleiotropy and heterogeneity in the causal effect estimates. A P-value less than 0.05 is typically interpreted as indicative of such effects. Clearing outliers can help reduce the impact of horizontal pleiotropy, after which the causality statistics can be re-run to obtain more accurate results. If significant horizontal pleiotropy and heterogeneity persist even after clearing the outliers, SNPs with a P-value less than 1 in the MR-PRESSO outlier test should be removed, followed by re-running the MR analysis. The results of the IVW model for random effects will be taken as the final causal effect if heterogeneity remains. The stability of the results was evaluated using the leave-one-out test, wherein each SNP was removed sequentially, and the IVW analysis was repeated to determine if any individual SNP significantly impacted the overall results.

## 3 Results

### 3.1 Description of the date

In the instrumental extraction stage, we searched for GWAS-significant SNPs using a function with a significance threshold set to p < 5 × 10^-8^ and p < 5 × 10^-6^ when necessary. R2 = 0.001 and kb = 10,000 were selected to exclude SNPs in strong LD. Subsequently, to perform MR, we harmonized the data since the effects of SNPs on outcome and exposure had to be reconciled relative to the same allele. Our harmonization process takes conservative action 2: To infer favorable strand alleles using allele frequencies for palindromes. Then, we removed the SNPs for incompatible alleles and SNPs being palindromic with intermediate allele frequencies. Finally, we used MR-PRESSO to remove outliers (ineligible SNPs) to correct for heterogeneity and horizontal pleiotropy. Ultimately, 15, 22, and 36 SNPs were included for MR analysis of the effect of three different occupational exposures on RA and 15, 24, and 36 SNPs to investigate the causal relationship with AS. The mean F-statistic value for JWS-RA, JWS-AS, was 42.48. Meanwhile, the average F-statistic values of JMP-RA, and JMP-AS, were 38,91. The mean F-statistic values of JSW-RA, JSW-AS, were 23.75. **Supplementary Table 8** provides information on the statistical efficacy and R² values. It is worth noting that the statistical effectiveness of certain studies may appear low due to the relatively limited sample size employed in those studies. The sample size plays a crucial role in determining the statistical power and precision of the analysis. In cases where the sample size is insufficient, the estimates may have wider confidence intervals and lower statistical significance. Nonetheless, it is essential to consider the overall body of evidence and the collective findings of the studies to draw broad conclusions.

### 3.2 Univariate Mendelian randomization of the causal relationship between occupational exposure and RA with AS

**Figure 2:**
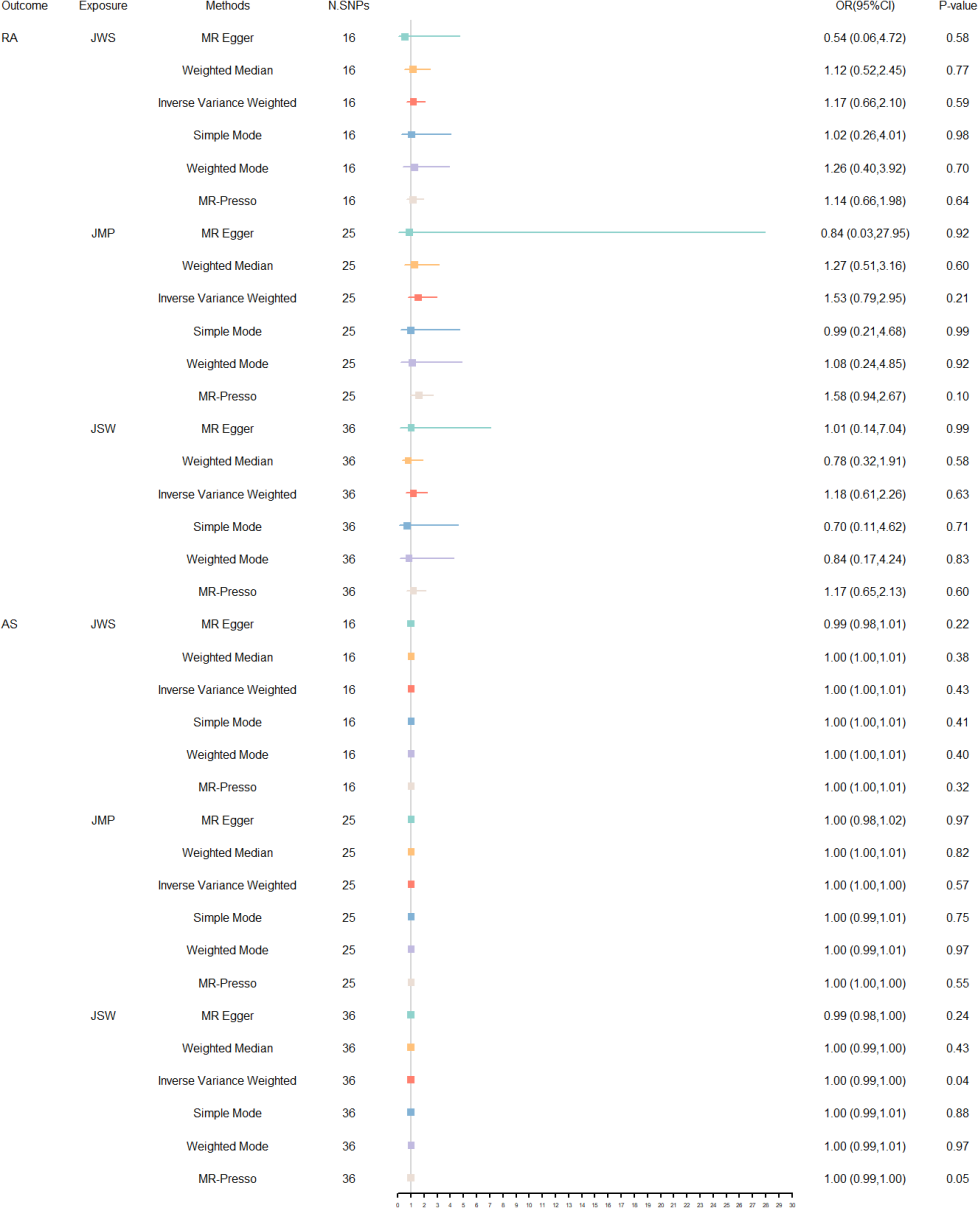
The forest plots of reversed UVMR analyses explored the relationship between jobs involving mainly walking or standing (JWS), jobs involving heavy manual or physical work (JMP), jobs involving shift work (JSW) on RA and AS on using different MR statistical models. The causal estimates are given as odds ratios (ORs) and 95% confidence intervals (CIs).

The MR estimates for the different methods are shown in **Figure 2**. Overall, no predicted causal relationships were observed between additional occupational exposure and RA, AS. Upon removal of all outliers, no significant heterogeneity was observed in the relationship between jobs involving mainly walking or standing and RA and AS. Utilizing 15 SNPs that were strongly linked to them, the analysis revealed no significant causal association with an elevated risk of RA [IVW: OR = 1.17, 95% CI = 0.66- 2.10 p = 0.59] and AS [IVW: OR = 1.00, 95% CI = 1.00- 1.01, p = 0.43]. Moreover, consistent results were shown by MR-Egger, weighted median, simple mode, and weighted mode methods. Additionally, in the absence of heterogeneity or pleiotropy, employing 22 and 24 SNPs associated with jobs involving heavy manual or physical work, no robust association was identified between this occupational exposure and the development of RA [IVW: OR = 1.53, 95% CI = 0.79- 2.95, p = 0.21] and AS [IVW: OR = 1.00, 95% CI = 1.00- 1.00, p = 0.57]. The number of SNPs at p < 5 × 10^-8^ was less than 10, so a more lenient threshold [p = 5 × 10^-6^] was used, and 36 SNPs involving shift work were finally identified. No causal link between jobs involving shift work and the prevalence of RA [IVW: OR = 1.18, 95% CI = 0.61- 2.26, p = 0.63] and AS [IVW: OR = 1.00, 95% CI = 0.99- 1.00, p = 0.04] was also discovered, even in the lack of heterogeneity or pleiotropy. Detailed results of the UVMR analysis are provided in **Supplementary Table 6**. In contrast, the sensitivity analysis of heterogeneity and pleiotropy of the original and adjusted MR analysis is shown in **Supplementary Table 7**. Additionally, **Supplementary Figures 3-11** presents scatter plots, leave-one-out stability tests, and funnel plots of the UVMR analysis results. No significant influence of SNPs impacting causality was observed. The results of statistical power and R² are available in **Supplementary Table 8**, and the statistical efficacy of some studies is low due to the relatively insufficient sample size.

### 3.3 Multivariate Mendelian randomization of the causal relationship between occupational exposure and RA with AS

To begin with, we omitted the effects of smoking, obesity, depression, infections, vitamin D deficiency from our analysis. We employed the IVW and MR-EGGER models to investigate the impact of three distinct work categories on RA and AS (**Supplementary Table 9**). The results demonstrated a causal link between JMP and the development of AS [MR-EGGER: OR = 1.01,95% CI =1.00-1.02, p = 0.02] (**Supplementary Figure 12**). However, this positive result was not statistically significant according to Bonferroni correction. Also, no causal relationship between the different operating states and RA and AS could be observed. Nevertheless, upon adjusting for the effects of obesity, smoking, depression, infection and vitamin D deficiency, our analysis found no evidence of a causal relationship between them. In addition, no causal relationship could be observed between occupational exposure and RA and AS in adjusted analysis (**Supplementary Figures 13, 14**). Information on heterogeneity and pleiotropy for the MVMR between occupational exposure to RA and AS was shown in **Supplementary Table 10**.

### 3.4 Bidirectional Mendelian randomization of the causal relationship between RA, AS and occupational exposure

To examine potential reverse causality between occupational exposures and RA or AS, in our study we performed a bidirectional MR analysis by exchanging exposure variables and outcome variables, which may help us to determine whether the observed correlation is solely due to exposure or whether there may be a reciprocal causal relationship (**Supplementary Figure 2**). Through reverse MR, we can explore whether certain diseases cause individuals to make different choices about their work environments or whether there are other factors that influence the choice of work environments, thus furthering our understanding of the interactions between diseases and work environments. Using 9 SNPs for RA-JWS, RA-JMP and RA- JSW, respectively, it was not shown that RA was significantly causally associated with JWS [IVW: OR = 1.00, 95% CI =0.99- 1.01, p =0.73], JMP [IVW: OR = 1.00, 95% CI =0.99- 1.01, p = 0.70], and JSW [IVW: OR = 0.99, 95% CI =0.99- 1.00, p = 0.16]. Also, after using 29 SNPs closely associated with AS-JWS, AS-JMP,AS-JSW, the results of TSMR showed no statistically significant causal relationship between AS and increased risk of JWS [IVW: OR =0.82, 95% CI =0.35- 1.92, p = 0.65], JMP [IVW: OR =0.87,95% CI =0.45- 1.70, p = 0.68], or JSW [IVW: OR =0.88, 95% CI=0.42- 1.85, p = 0.74]. The results in AS-JSW showed a case of horizontal pleiotropy in the MR Egger intercept test, however, MR-PRESSO did not detect the phenomenon. We believe that this result may be attributed to insufficient sample size or variability between methods, as well as the possibility of false positives. We would overall take several other results as a reference and interpret them with caution (**Supplementary Table 7**).

## 4 Discussion

In this study, we utilized UVMR and MVMR to investigate the causal relationship between occupational exposure to RA and AS using pooled GWAS data. In detail, we could not demonstrate a bidirectional causal relationship between occupational exposure and RA together with AS by using MR methods. Our UVMR analysis revealed no evidence supporting a causal link between specific types of jobs, including those requiring prolonged standing or walking, heavy manual labor or physical exertion, shift work, and an increased risk of RA and AS. The unadjusted MVMR result may show an independent causal relationship between JMP and the occurrence of AS, but Bonferroni Correction rejected it. The five covariates included in the MVMR (smoking, obesity, depression, infection and vitamin D deficiency) all play an important role in the pathogenesis of RA and AS (39–43). Smoking is known to trigger an inflammatory response that promotes joint inflammation and tissue damage in patients with RA and AS. On the other hand, obesity contributes to chronic low-grade inflammation by releasing pro-inflammatory adipokines that exacerbate disease severity. Psychological stress and depression can negatively affect the immune system, leading to a dysregulated inflammatory response and potential disease flare-ups. In genetically susceptible individuals, infections can act as triggers for RA and AS, inducing an autoimmune response to joint tissues. Finally, vitamin D deficiency has a slightly different pathogenesis and progression in different RA groups but is associated with disruption of vitamin D signaling. By adjusting for these covariates in MVMR analyses, we aimed to mitigate potential confounding effects and more accurately assess the causal relationship between occupational exposure and RA/AS. However, our MVMR adjusting for smoking, obesity, depression, infection and vitamin D deficiency showed that JMP was not causally related to AS for these characteristics.

Although we have not demonstrated a causal relationship between specific occupational settings on RA and AS, it is undeniable that environmental factors do have an impact on RA and AS. The results of our adjusted MVMR study suggest that jobs involving heavy manual or physical work may not be the etiology of AS, which is opposed to the results of several studies (44, 45). These observational studies concluded that there is a causal association between heavy manual or physical work and AS. The mechanisms by which heavy physical work may lead to AS have yet to elucidate fully, but some hypotheses may be involved. First, prolonged serious physical work exposes the skeletal system to chronic mechanical stress, leading to joint and ligament damage and cell death, which releases autoantigens that may be recognized as foreign by the immune system, and this triggers an aggressive immune response resulting in inflammation and tissue damage, which are the main features of AS (46, 47). Subsequently, immune cells and factors promote tissue repair, but their persistence beyond the healing phase may trigger ongoing inflammation and damage, leading to the progression of AS. Second, engaging in prolonged heavy physical work may lead to a state of physical and mental stress. Stress can increase the levels of circulating inflammatory markers, enhance the intensity of the acute inflammatory response, and ultimately stimulate low-grade systemic inflammation, which can cause inflammation and damage to joints and ligaments, thus further facilitating the aggressiveness of the immune system and creating a vicious cycle that exacerbates the development and progression of AS (48). In addition, when performing heavy physical work, the body uses much energy and produces metabolic waste products such as lactic acid, urea, and uric acid. If these metabolites are not excreted promptly, they can accumulate in the body. Studies have shown that increased blood uric acid and the formation of urate crystals may have a facilitative role in the development of chronic inflammation associated with a variety of diseases, and urate crystals may also induce the activation of inflammatory vesicles, and these inflammatory responses may promote the development of AS. Furthermore, it has been suggested that imaging grading of the sacroiliac joint in patients with medial spondyloarthritis is closely associated with the progression of urate crystal deposition in the pelvic region, so the development of AS may be metabolically related to physical exertion (49, 50). Although some studies have proposed that strenuous physical work may increase the possibility of developing RA (19, 51). However, in line with our findings, some reports suggest that physical labor may be connected with a diminished risk of RA and lower levels of inflammation (52–54), implying that proper physical labor levels might serve as a protective factor for RA. This could be attributed to the fact that labor can potentially decrease systemic inflammation (55, 56) and regulate the immune system (57–59), and these studies further solidify our conclusions. Fewer studies have been conducted on the relationship between shift work on RA and AS, and our findings suggest no association between the two, which is consistent with the conclusions reached in an observational study (21). This observational study concluded that melatonin production is reduced in long-term night shift workers, and some evidence suggests a disease-promoting role for melatonin in RA, making long-term night shifts protective against RA. The exact cause of AS is unknown, and the causal relationship between shift work and it is unclear. Although our MR study concluded that shift work was not associated with the development of AS, environmental factors may still influence its incidence, and thus some scholars have suggested that work stress due to shift work is considered a potential causative factor for AS (45). The relationship between occupational exposures involving night work and rheumatic diseases appears complex and unclear, necessitating further studies to elucidate it. No studies to date seem to suggest that excessive walking or standing can lead to RA or AS, which is consistent with our findings. However, lower extremity fatigue associated with excessive walking or standing is a risk factor for RA, but the quality of evidence in this literature is insufficient to support this conclusion. Therefore, our study demonstrates that excessive walking or standing is not a cause of RA or AS.

We interpreted our findings with great caution, attributing them to the limitations of the study design. Although our study elucidated the relationship between specific occupational exposures and the risk of RA and AS, it was insufficient to determine whether environmental factors are more substantial than exposure-related genetic variants. In examining the influence of environmental factors on the development of RA and AS, we first realized the important role of genetic factors in determining individual susceptibility. In previous studies, specific genetic markers (e.g., HLA-DRB1 and HLA-B27) have been widely shown to be significant risk factors for RA and AS. The presence of these genetic variants increases the likelihood of developing these diseases. Identification of these genetic markers highlights the impact of specific genetic variants on disease susceptibility and provides insights into the underlying mechanisms. However, the impact of genetic variants is not limited to association with specific occupational exposures. They contribute to an individual’s overall genetic background and can modulate their response to environmental stimuli. Therefore, it is important to recognize that genetic variants may alter an individual’s tolerance or response to certain environmental factors, thereby influencing the development and progression of RA and AS. Also, through GWAS, we have identified other genetic variants and polymorphisms associated with RA and AS risk. These genetic markers are involved in processes such as immune regulation, inflammation, and tissue remodeling, providing valuable insights into the pathogenesis of these diseases. Therefore, when considering the “strength” of environmental factors relative to the genetic variants associated with occupational exposures, it is important to recognize the significant contribution of genetic factors to disease susceptibility and to understand their interactions with environmental stimuli.

In addition, there may be interactions between environmental factors and genetic variation. Genetic background may influence an individual’s sensitivity and response to environmental factors. This means that specific genetic variants may make some individuals more sensitive to specific occupational exposures while others are less susceptible. This interaction complicates the role of environmental factors and genetic variation in disease pathogenesis. For example, specific genetic variants may influence the effect of smoking on RA risk. Smokers with specific genetic variants may be more likely to develop RA than non-smokers or those without these genetic variants. This suggests that genetic factors influence the impact of smoking on the development of RA. Similarly, environmental factors such as obesity, depression, infections, and vitamin deficiencies (covariates of MVMR) may also be influenced by genetic variants. These genetic variants may affect an individual’s metabolism of certain substances, modulate immune responses, or influence inflammatory pathways, thereby affecting the overall risk of RA and AS. Understanding the interaction of genes and environmental factors is critical to understanding the heterogeneity, severity, and treatment response of RA and AS disease manifestations in patients.

Taken together, our findings suggest that both environmental factors and genetic variants play important roles in the pathogenesis of RA and AS and may interact in different ways. It may be difficult to compare their “strengths” because they play different roles in disease pathogenesis. Rather than simply comparing them, we should consider them to better understand their relative contributions in the pathogenesis of RA and AS.

Our findings provide new insights into the genetic basis of RA and AS, particularly the role of occupational factors in their pathogenesis, which may have important implications for occupational choices and health. For occupational physicians, our study provides valuable guidance in understanding the impact of specific occupational exposures on RA and AS. By discussing the work environment with patients and providing accurate information, physicians can provide comprehensive medical advice during diagnosis and treatment, reducing patient concerns and misunderstandings. Early prevention and timely intervention can be achieved by proactively asking patients about their occupational background, enabling timely identification and management of occupation-related health problems. In addition, our findings highlight the importance of improving the work environment to minimize the risk of developing occupationally related diseases, with occupational physicians playing a crucial role in formulating health and safety policies and enforcing standards. For workers, our study empowers them to make informed career choices by providing insights into the potential association between specific occupational exposures and RA/AS. Workers can take appropriate precautions and use personal protective measures to minimize the health risks associated with their work environment. In addition, our findings encourage workers to advocate for better working conditions and rights. By dialoguing with employers and participating in policy development, workers can create safer and healthier work environments, reduce the risk of occupational diseases and increase job satisfaction.

## 5 Limitation

This study has several limitations that need to be addressed in future research. Firstly, the use of genetic IVs may not be specific to occupational exposure, as this study only applied statistical methods and did not incorporate biological processes. Secondly, the majority of genome-wide association study data used in this study were derived from individuals of European descent, which may limit the generalizability of the findings to other populations with different lifestyles. Thirdly, subjective awareness of occupational exposure differs from objective measures, and there may be variations in the interpretation of occupational exposure and work intensity across different populations. Lastly, some risk factors for RA and AS remain unknown, and the exact biological function of many genetic variants is still unclear, warranting further investigation into the pathophysiological relationship between occupational exposure and RA and AS. These limitations may have implications for the reliability and generalizability of the study results. Future studies should aim to minimize the impact of these limitations by adopting methods such as increasing the sample size and proportion of cases in the sample to improve statistical power, conducting more interventions and cross-sectional studies to explore the relationship between occupational exposure and RA with AS in real-world settings, and incorporating additional MR methods to reduce discrepancies in causality inference. Addressing these limitations could facilitate progress and development in the RA and AS-related research field, improving treatment and management strategies for those affected by the condition. Notably, the significant variation in the causality of results across MR methods suggests the need for multiple MR study methods. In addition, better GWAS, as well as more types of rheumatism, are needed to clarify the causal relationship between them. The UVMR results differed significantly from the MVMR results, indicating that MVMR is necessary to correct confounding factors. Therefore, further research is required to explore the complex underlying mechanisms of the associations between occupational exposure and RA with AS. Finally, replication on an independent dataset would further improve the robustness and generalizability of the findings. However, we were unable to conduct replication analyses due to the lack of independent datasets that met the necessary criteria.

## 6 Conclusions

Our results showed that neither UVRM nor MVMR analyses demonstrated a causal relationship between specific job exposures and the progression of RA and AS. Further studies are needed to understand the causal pathways of the complex interactions between environmental and genetic factors before RA and AS.

## Data availability statement

The original contributions presented in the study are included in the article/**Supplementary Material**. Further inquiries can be directed to the corresponding author.

## Author contributions

Conceptualization, KD and SM-L. Software, JZ-H. Writing—original draft preparation, KD and AL. Writing—review and editing, CY-Z. Supervision, SM-L and RG. All authors contributed to the article and approved the submitted version.
