# Supplementary material for: Causality of occupational exposure on rheumatoid arthritis and ankylosing spondylitis: a two-sample mendelian randomization study
Source: Front Immunol. 2023 Oct 2;14:1223810. doi: 10.3389/fimmu.2023.1223810 (PMC10577282; doi:10.3389/fimmu.2023.1223810)
Supplement: Supplementary file 1 [file DataSheet_1.docx]

**Supplementary Figure 1.** Flowchart of the number of people included in each phase of the study and the reasons for exclusion. JWS: Jobs involving mainly walking or standing; JMP: Jobs involving heavy manual or physical work; JSW: Jobs involving shift work. RA: Rheumatoid arthritis; AS: Ankylosing Spondylitis.

**Supplementary Figure 2.** The forest plots of reversed UVMR analyses explored the relationship between RA and AS on jobs involving mainly walking or standing (JWS), jobs involving heavy manual or physical work (JMP), jobs involving shift work (JSW) using different MR statistical models. The causal estimates are given as odds ratios (ORs) and 95% confidence intervals (CIs).

**Supplementary Figure 3.** Scatter plots of causal estimates of jobs involving mainly walking or standing (JWS). The slope of each line corresponds to the estimated MR effect in different models, including the conventional Inverse Variance Weighted, MR Egger, Simple Mode, Weighted Mode, and Weighted Median. The effect of A: Rheumatoid arthritis; B: Ankylosing spondylitis.

**Supplementary Figure 4.** Scatter plots of causal estimates of jobs involving heavy manual or physical work (JMP). The slope of each line corresponds to the estimated MR effect in different models, including the conventional Inverse Variance Weighted, MR Egger, Simple Mode, Weighted Mode, and Weighted Median. The effect of A: Rheumatoid arthritis; B: Ankylosing spondylitis.

**Supplementary Figure 5.** Scatter plots of causal estimates of jobs involving shift work (JSW). The slope of each line corresponds to the estimated MR effect in different models, including the conventional Inverse Variance Weighted, MR Egger, Simple Mode, Weighted Mode, and Weighted Median. The effect of A: Rheumatoid arthritis; B: Ankylosing spondylitis.

**Supplementary Figure 6.** Leave-one-out stability tests causal estimates of exposure (Jobs involving mainly walking or standing) on outcomes. Calculate the MR results of the remaining IVs after removing the IVs one by one. The effect of A: Rheumatoid arthritis; B: Ankylosing spondylitis**.**

**Supplementary Figure 7.** Leave-one-out stability tests causal estimates of exposure (Jobs involving heavy manual or physical work) on outcomes. Calculate the MR results of the remaining IVs after removing the IVs one by one. The effect of A: Rheumatoid arthritis; B: Ankylosing spondylitis**.**

**Supplementary Figure 8.** Leave-one-out stability tests causal estimates of exposure (Jobs involving shift work) on outcomes. Calculate the MR results of the remaining IVs after removing the IVs one by one. The effect of A: Rheumatoid arthritis; B: Ankylosing spondylitis

**Supplementary Figure 9.** Funnel plot of single SNP analysis of exposure (Jobs involving mainly walking or standing) by Inverse Variance Weighting and MR EGGER on outcomes. Effects of A: Rheumatoid arthritis; B: Ankylosing spondylitis.

**Supplementary Figure 10.** Funnel plot of single SNP analysis of exposure (Jobs involving heavy manual or physical work) by Inverse Variance Weighting and MR EGGER on outcomes. Effects of A: Rheumatoid arthritis; B: Ankylosing spondylitis.

**Supplementary Figure 11.** Funnel plot of single SNP analysis of exposure (Jobs involving shift work) by Inverse Variance Weighting and MR EGGER on outcomes. Effects of A: rheumatoid arthritis; D: ankylosing spondylitis.

**Supplementary Figure 12.** The forest plot of the multi-variable MR analysis. Causal estimates for the effect of working status on arthritis were given as odds ratio (OR) and 95% confidence intervals (CIs). JWS: Jobs involving mainly walking or standing; JMP: Jobs involving heavy manual or physical work; JSW: Jobs involving shift work. RA: Rheumatoid arthritis; AS: Ankylosing Spondylitis.

**Supplementary Figure 13.** The forest plot of the multi-variables IVW model. Causal estimates for the effect of working status on RA and AS were given as odds ratio (OR) and 95% confidence intervals (CIs) and were adjusted by obesity, smoking, depression, infection, and Vitamin D deficiency. JWS: Jobs involving mainly walking or standing; JMP: Jobs involving heavy manual or physical work; JSW: Jobs involving shift work. RA: Rheumatoid arthritis; AS: Ankylosing Spondylitis.

**Supplementary Figure 14.** The forest plot of the multi-variables MR-Egger. Causal estimates for the effect of working status on RA and AS were given as odds ratio (OR) and 95% confidence intervals (CIs) and were adjusted by obesity, smoking, depression, infection, and Vitamin D deficiency. JWS: Jobs involving mainly walking or standing; JMP: Jobs involving heavy manual or physical work; JSW: Jobs involving shift work. RA: Rheumatoid arthritis; AS: Ankylosing Spondylitis.


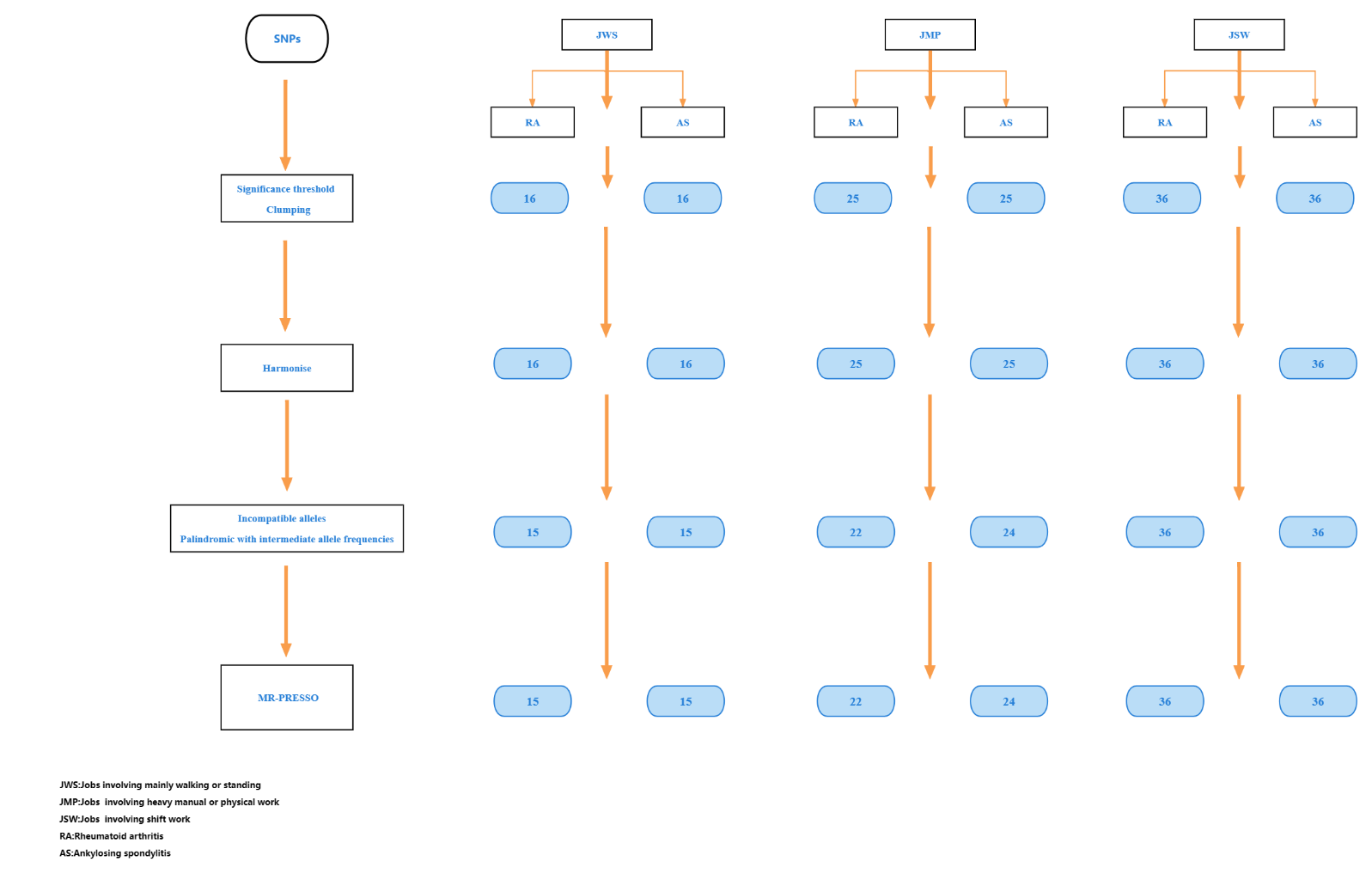


**Supplementary Figure 1.** Flowchart of the number of people included in each phase of the study and the reasons for exclusion. JWS: Jobs involving mainly walking or standing; JMP: Jobs involving heavy manual or physical work; JSW: Jobs involving shift work. RA: Rheumatoid arthritis; AS: Ankylosing Spondylitis.


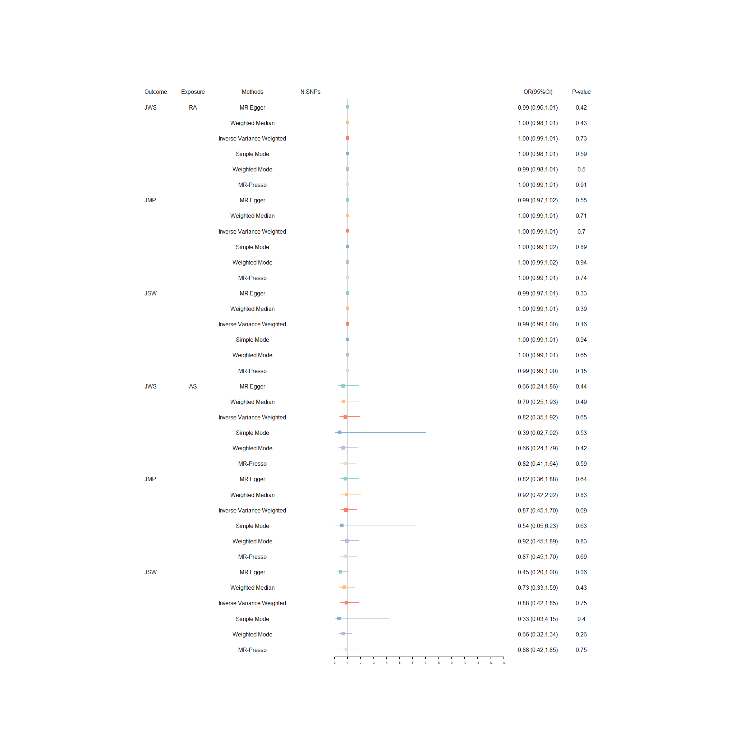


**Supplementary Figure 2.** The forest plots of reversed UVMR analyses explored the relationship between RA and AS on jobs involving mainly walking or standing (JWS), jobs involving heavy manual or physical work (JMP), jobs involving shift work (JSW) using different MR statistical models. The causal estimates are given as odds ratios (ORs) and 95% confidence intervals (CIs).

**
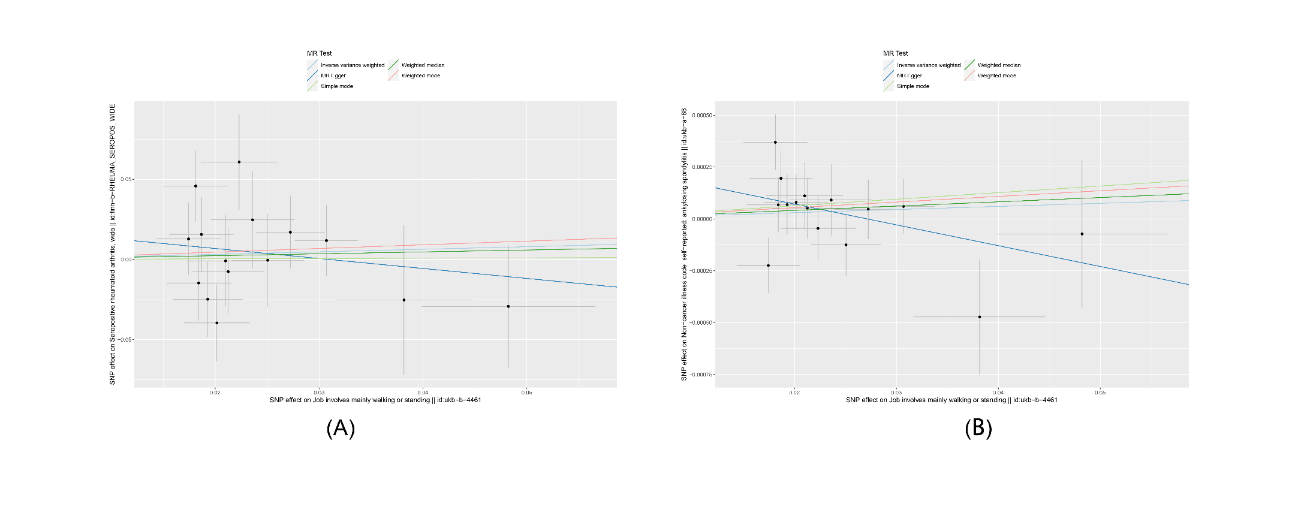
**

**Supplementary Figure 3.** Scatter plots of causal estimates of jobs involving mainly walking or standing (JWS). The slope of each line corresponds to the estimated MR effect in different models, including the conventional Inverse Variance Weighted, MR Egger, Simple Mode, Weighted Mode, and Weighted Median. The effect of A: Rheumatoid arthritis; B: Ankylosing spondylitis


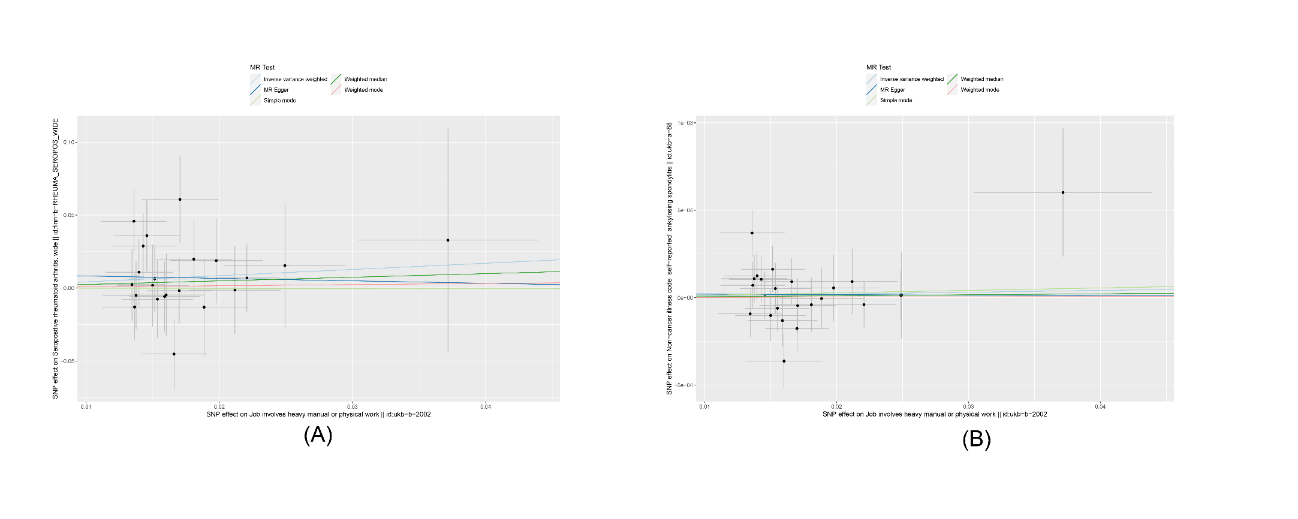


**Supplementary Figure 4.** Scatter plots of causal estimates of jobs involving heavy manual or physical work (JMP). The slope of each line corresponds to the estimated MR effect in different models, including the conventional Inverse Variance Weighted, MR Egger, Simple Mode, Weighted Mode, and Weighted Median. The effect of A: Rheumatoid arthritis; B: Ankylosing spondylitis.


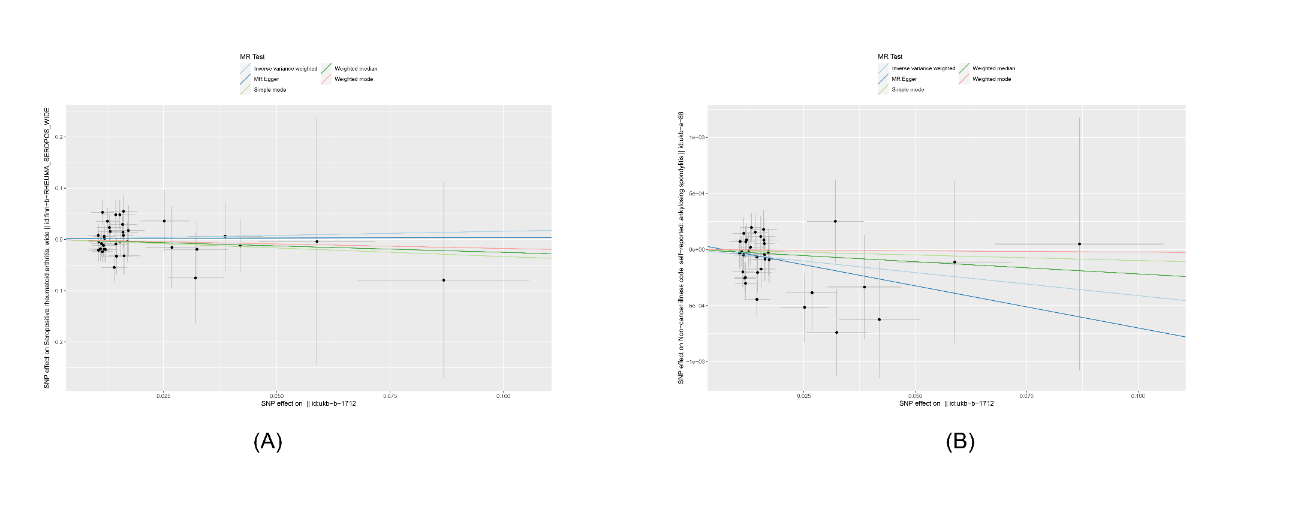


**Supplementary Figure 5.** Scatter plots of causal estimates of jobs involving shift work (JSW). The slope of each line corresponds to the estimated MR effect in different models, including the conventional Inverse Variance Weighted, MR Egger, Simple Mode, Weighted Mode, and Weighted Median. The effect of A: Rheumatoid arthritis; B: Ankylosing spondylitis.


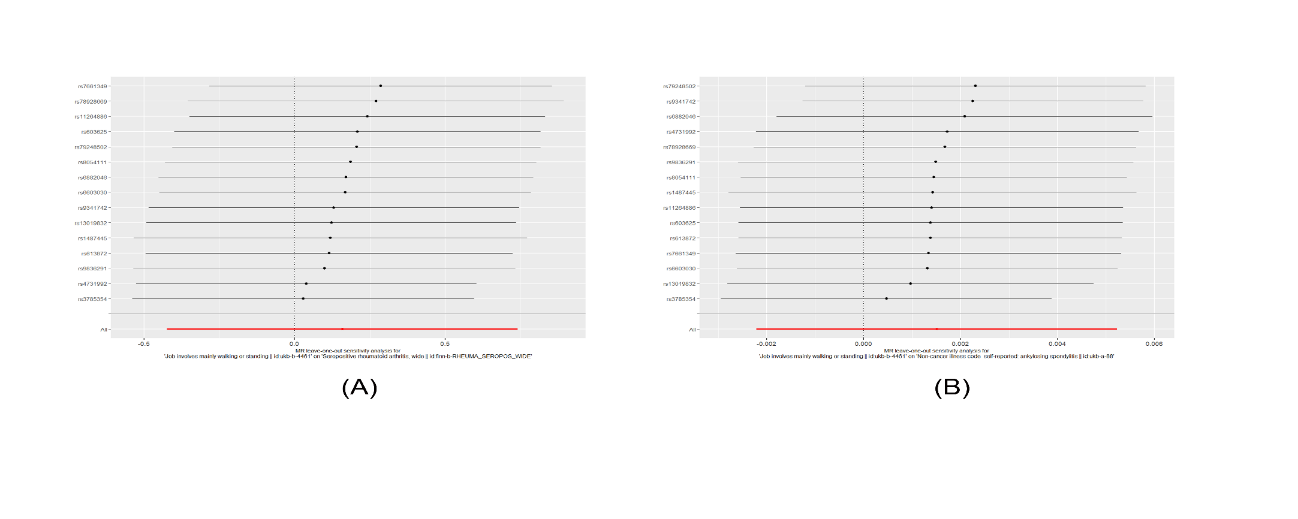


**Supplementary Figure 6.** Leave-one-out stability tests causal estimates of exposure (Jobs involving mainly walking or standing) on outcomes. Calculate the MR results of the remaining IVs after removing the IVs one by one. The effect of A: Rheumatoid arthritis; B: Ankylosing spondylitis.

**
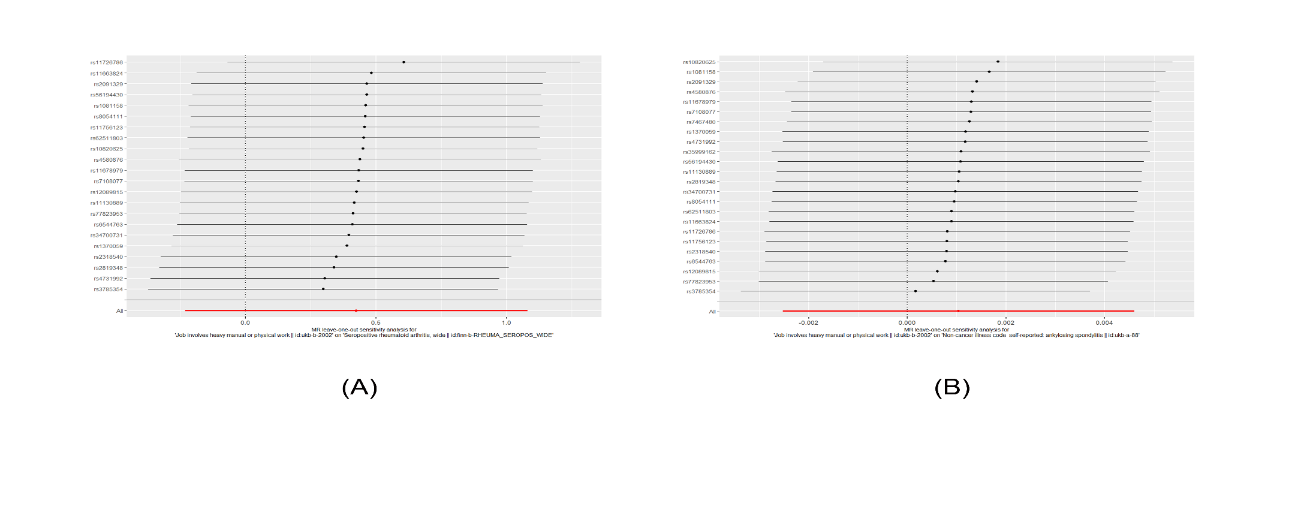
**

**Supplementary Figure 7.** Leave-one-out stability tests causal estimates of exposure (Jobs involving heavy manual or physical work) on outcomes. Calculate the MR results of the remaining IVs after removing the IVs one by one. The effect of A: Rheumatoid arthritis; B: Ankylosing spondylitis.

**
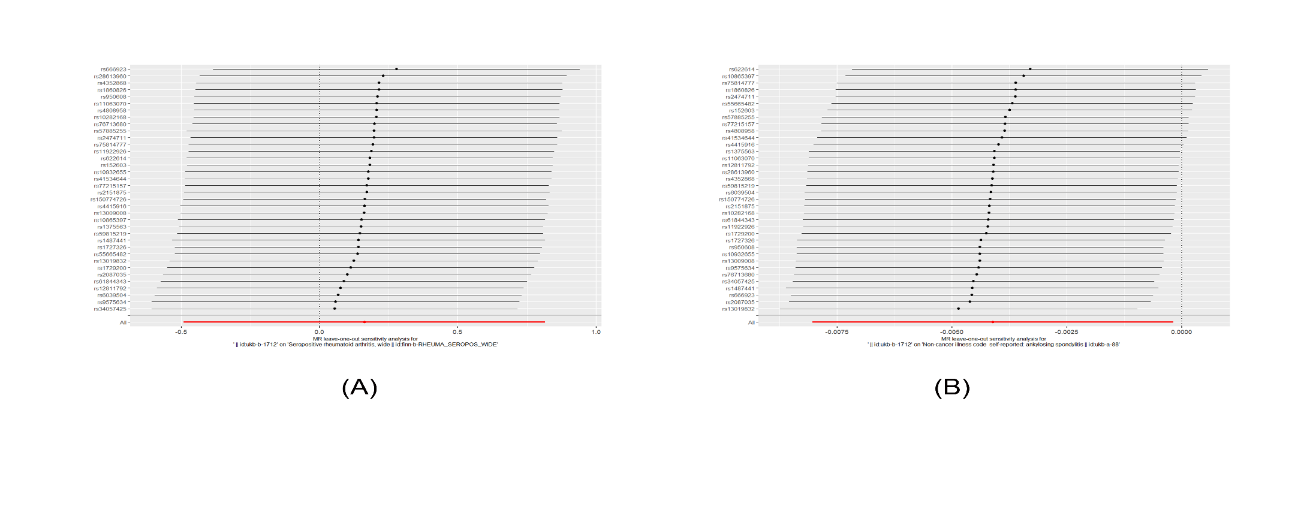
**

**Supplementary Figure 8.** Leave-one-out stability tests causal estimates of exposure (Jobs involving shift work) on outcomes. Calculate the MR results of the remaining IVs after removing the IVs one by one. The effect of A: Rheumatoid arthritis; B: Ankylosing spondylitis.


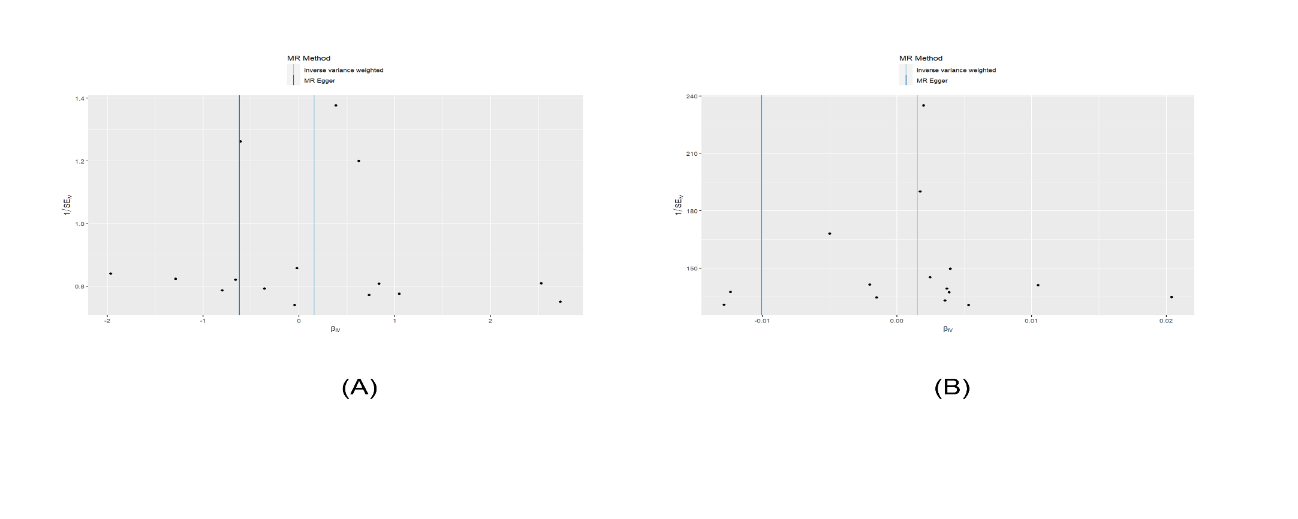


**Supplementary Figure 9.** Funnel plot of single SNP analysis of exposure (Jobs involving mainly walking or standing) by Inverse Variance Weighting and MR EGGER on outcomes. Effects of A: Rheumatoid arthritis; B: Ankylosing spondylitis.


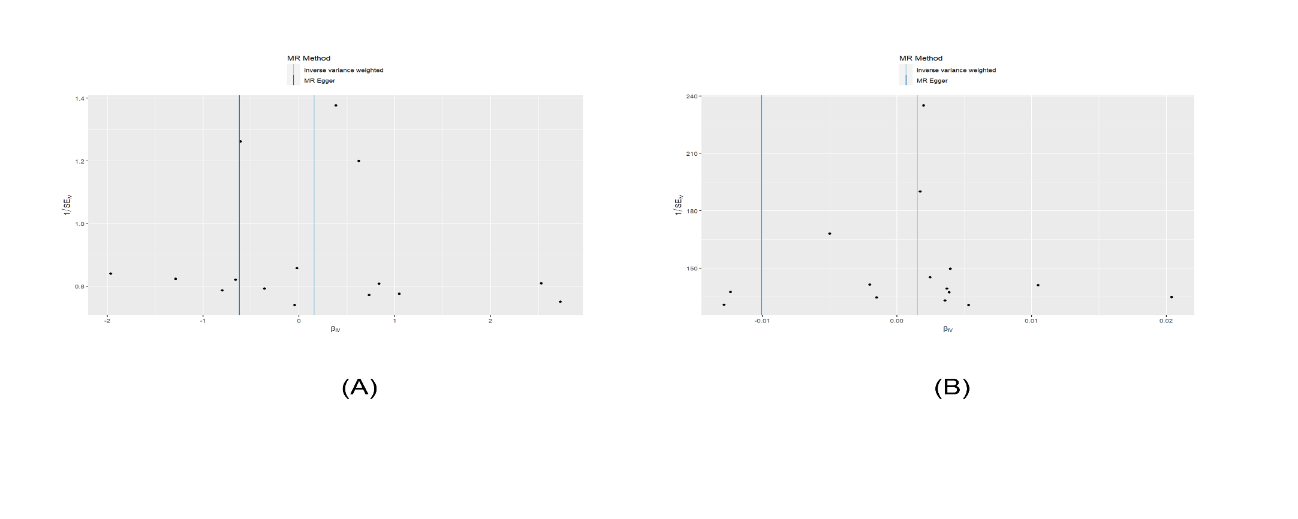


**Supplementary Figure 10.** Funnel plot of single SNP analysis of exposure (Jobs involving heavy manual or physical work) by Inverse Variance Weighting and MR EGGER on outcomes. Effects of A: Rheumatoid arthritis; B: Ankylosing spondylitis.


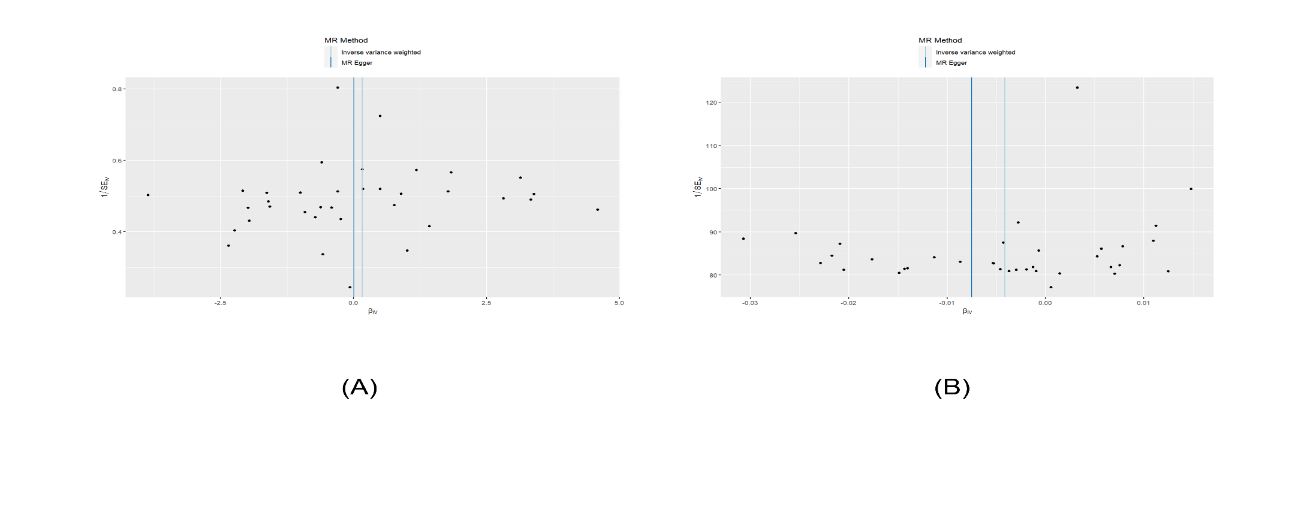


**Supplementary Figure 11.** Funnel plot of single SNP analysis of exposure (Jobs involving shift work) by Inverse Variance Weighting and MR EGGER on outcomes. Effects of A: Rheumatoid arthritis; B: Ankylosing spondylitis.


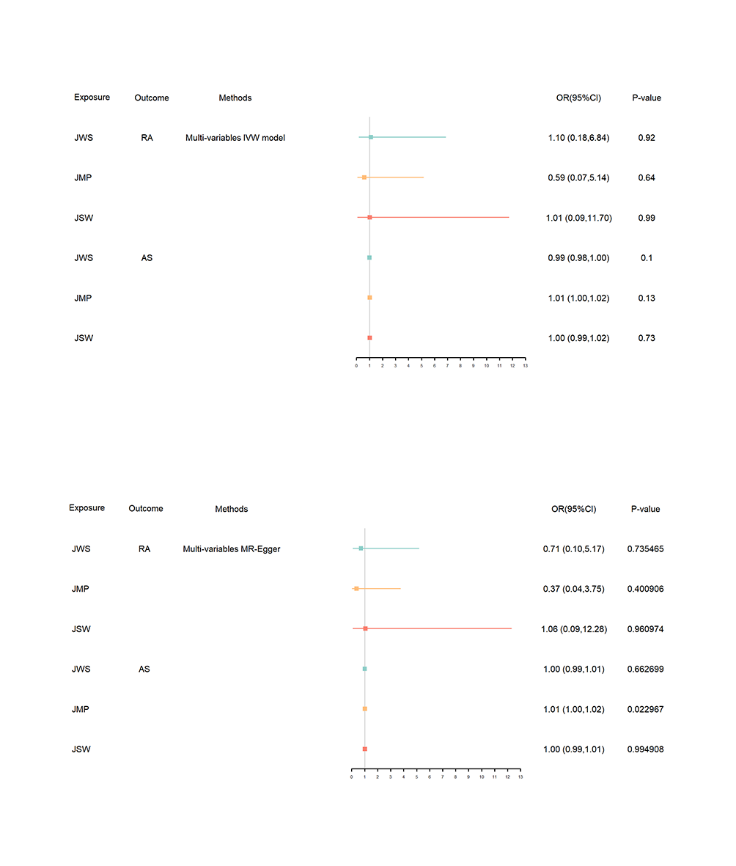


**Supplementary Figure 12.** The forest plot of the multi-variable MR analysis. Causal estimates for the effect of working status on arthritis were given as odds ratio (OR) and 95% confidence intervals (CIs). JWS: Jobs involving mainly walking or standing; JMP: Jobs involving heavy manual or physical work; JSW: Jobs involving shift work. RA: Rheumatoid arthritis; AS: Ankylosing Spondylitis.


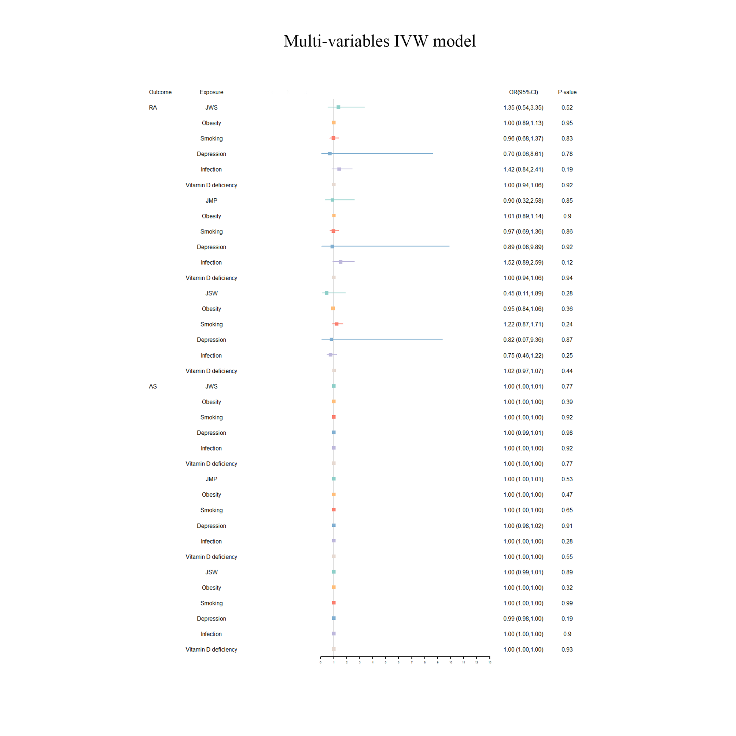


**Supplementary Figure 13.** The forest plot of the multi-variables IVW model. Causal estimates for the effect of working status on RA and AS were given as odds ratio (OR) and 95% confidence intervals (CIs) and were adjusted by obesity, smoking, depression, infection, and Vitamin D deficiency. JWS: Jobs involving mainly walking or standing; JMP: Jobs involving heavy manual or physical work; JSW: Jobs involving shift work. RA: Rheumatoid arthritis; AS: Ankylosing Spondylitis.


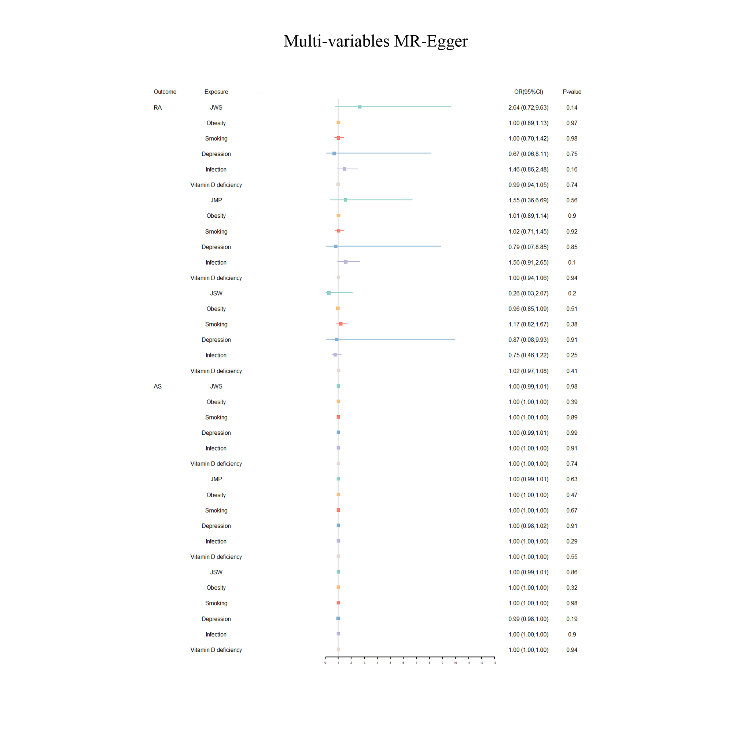


**Supplementary Figure 14.** The forest plot of the multi-variables MR-Egger. Causal estimates for the effect of working status on RA and AS were given as odds ratio (OR) and 95% confidence intervals (CIs) and were adjusted by obesity, smoking, depression, infection, and Vitamin D deficiency. JWS: Jobs involving mainly walking or standing; JMP: Jobs involving heavy manual or physical work; JSW: Jobs involving shift work. RA: Rheumatoid arthritis; AS: Ankylosing Spondylitis.
